# Markedly Exophytic Hypertrophic Lichen Planus in a Patient With Ichthyosis Vulgaris

**DOI:** 10.7759/cureus.73762

**Published:** 2024-11-15

**Authors:** Jack Catoe, Andrew Siref, Christopher Huerter

**Affiliations:** 1 Dermatology, Creighton University School of Medicine, Omaha, USA; 2 Pathology, Creighton University School of Medicine, Omaha, USA

**Keywords:** atypical lichen planus, hyperkeratosis, hypertrophic lichen planus, icthyosis vulgaris, skin of color

## Abstract

Hypertrophic lichen planus (HLP) is an idiopathic inflammatory condition characterized by hyperkeratotic plaques or nodules, typically occurring bilaterally on the wrists, ankles, or lower extremities. This variant of lichen planus is more common among African-American patients and occupies a broad differential with other keratotic skin conditions, some of which are malignant, making recognition and accurate diagnosis essential. We present an unusual case of a 49-year-old African-American woman with four markedly exophytic, horn-like lesions on her shins, ultimately diagnosed as HLP. Her medical history was also notable for ichthyosis vulgaris. Although no known interaction exists between HLP and ichthyosis vulgaris, the combination of these conditions may have contributed to intense pruritus, leading to increased excoriation. This excoriation could have influenced the unusual growth pattern, as mechanical disruption is associated with increased keratin production in hyperkeratotic conditions.

## Introduction

Lichen planus (LP) is a chronic, idiopathic inflammatory condition that can affect both cutaneous and mucosal surfaces [1,2]. Recent research suggests an autoimmune component in LP pathogenesis, with T-cell cytokine release in affected tissues [1]. Cytotoxic CD8+ T cells, both resident in the skin and circulating, are believed to release interferon-γ and tumor necrosis factor-α, causing direct tissue damage [1]. Environmental factors, including viral infections (particularly hepatitis C), medications such as ACE inhibitors, beta-blockers, and anticonvulsants, and contact allergens, have been implicated in the development of LP [1,2].

The condition derives its name from its lichen-like appearance, resembling flat-topped growths of algae or moss [2]. The classic presentation of cutaneous LP is described by the “Six P’s”: planar, polygonal, pruritic, purple, plaques, and papules [2]. A distinctive feature of both mucosal and cutaneous LP is Wickham striae, a lacy pattern of gray-white lines overlying the lesions [1-3]. LP has several subtypes, each with unique presentations [2]. One such subtype is hypertrophic LP (HLP), also referred to as LP verrucosus [1-3]. HLP typically presents as gray, hyperkeratotic plaques, often on the anterior lower legs, ankles, or wrists bilaterally, and is commonly associated with pruritus and pain [1,2]. HLP is most prevalent in individuals aged 40-70, with recent studies indicating a higher prevalence among the elderly than previously recognized [1,4]. While LP variants are more common in patients of European descent, HLP is more frequent in patients of African descent and other populations with darker skin tones [1,4]. The condition predominantly affects females, with a female-to-male ratio estimated at 1.5:1 to 3:1 [1,4].

Diagnosis is typically based on clinical appearance and features [1,3]. Histologic examination showing hyperkeratosis and interface dermatitis may support the diagnosis, though HLP can be difficult to confirm through biopsy alone, as its characteristic features may not always be present [1,4]. The classic histologic presentation of cutaneous LP includes a band of neutrophilic infiltrate through the dermal-epidermal junction [1,2]. In HLP, infiltration is often concentrated near the tips of rete ridges, with eosinophils possibly present [1]. Biopsy can also help exclude other, more serious conditions.

The differential diagnosis of HLP is broad, encompassing squamous cell carcinoma (SCC), keratoacanthoma, and other hyperkeratotic conditions [1,2]. Accurate diagnosis and differentiation from similar conditions are essential for the timely initiation of appropriate therapy and to avoid unnecessary invasive interventions.

## Case presentation

**Figure 1:**
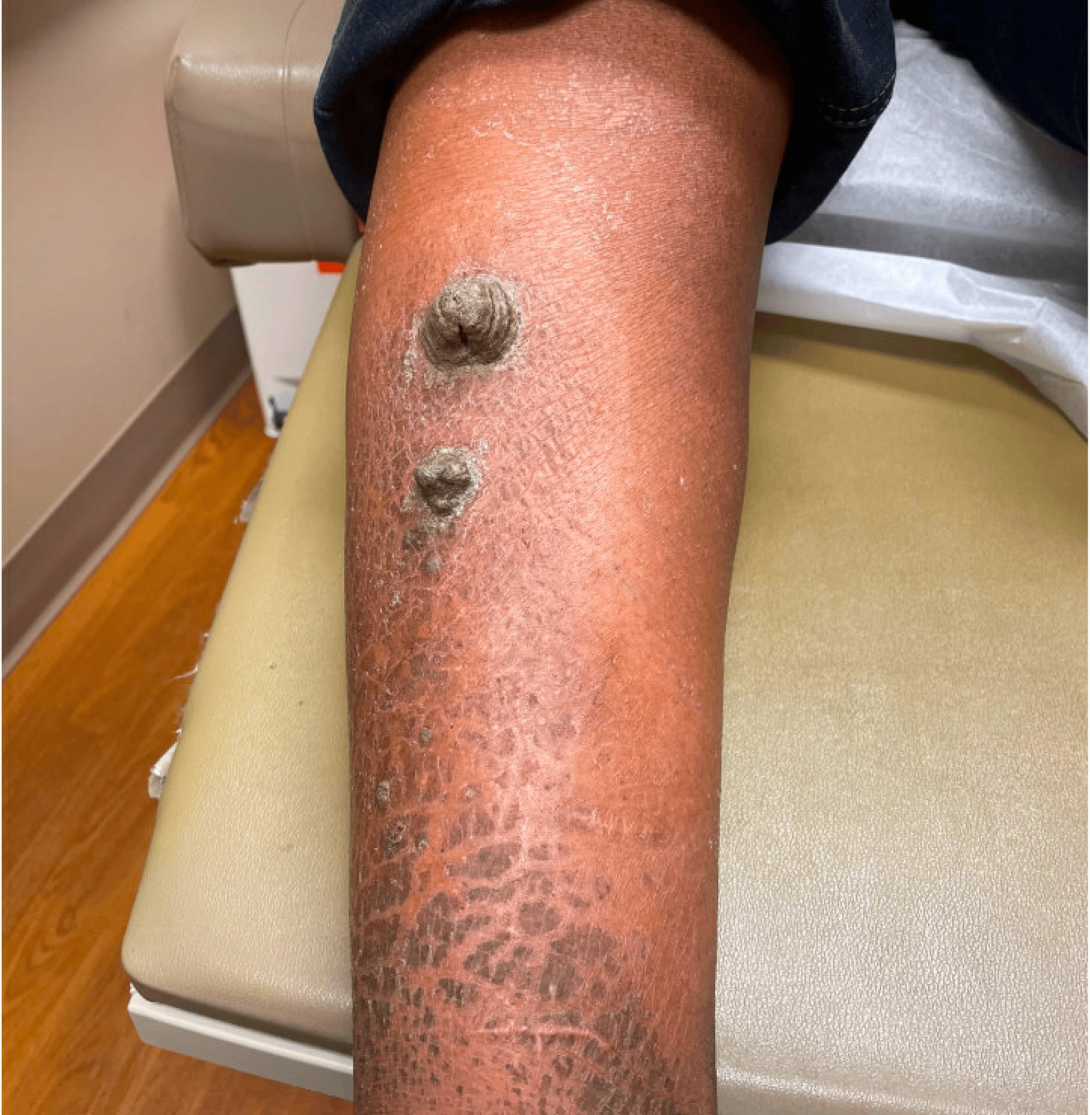
Two well-defined, exophytic gray plaques with diffuse dryness and scaling on the right lower leg

**Figure 2:**
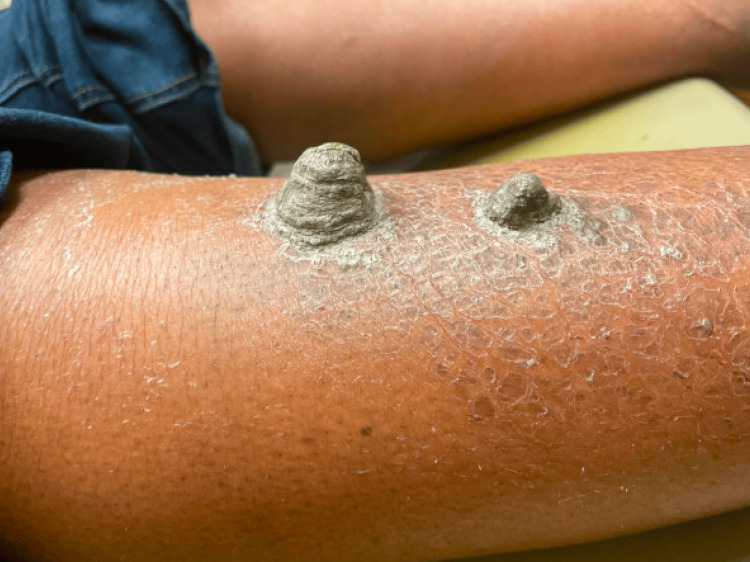
Side view of plaques on the right pretibial area, measuring 4.4 × 3.5 × 3.5 cm (left) and 1.8 × 2 × 2 cm (right)

A 49-year-old African-American woman with a medical history notable for ichthyosis vulgaris presented to the clinic with four markedly exophytic, gray, hyperkeratotic plaques in a bilateral distribution on the anterior lower legs (Figure 1, Figure 2). The patient reported intense pruritus and tenderness associated with the lesions. The largest lesion protruded 4.4 cm from the right shin. Severe xerosis and ichthyosis were noted on the skin underlying the plaques, along with multiple smaller hyperkeratotic papules on the distal shins. No other cutaneous or mucosal lesions were observed during the examination.

**Figure 3:**
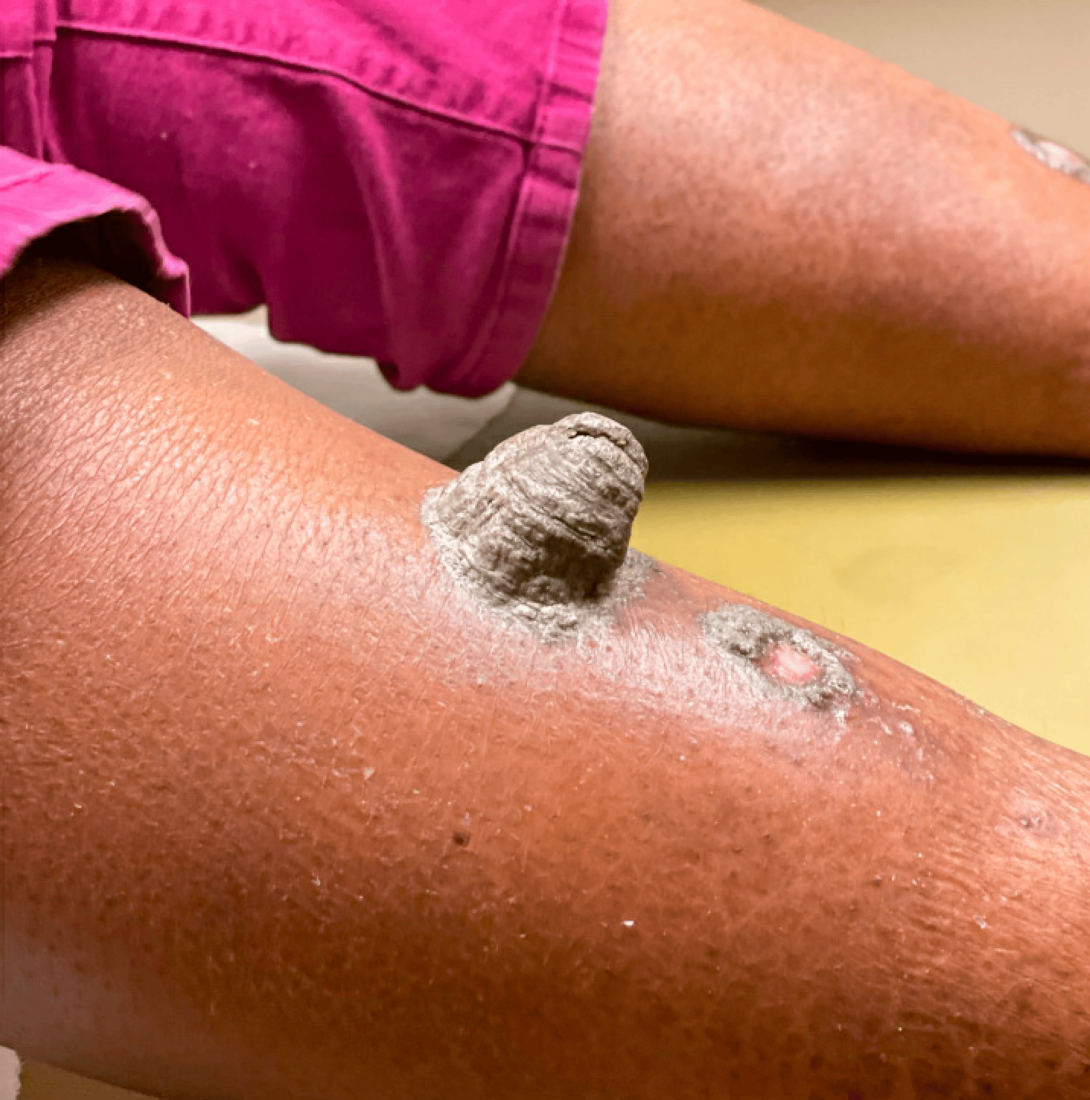
Right pretibial plaques at follow-up visit, after the first shave biopsy (right)

**Figure 4:**
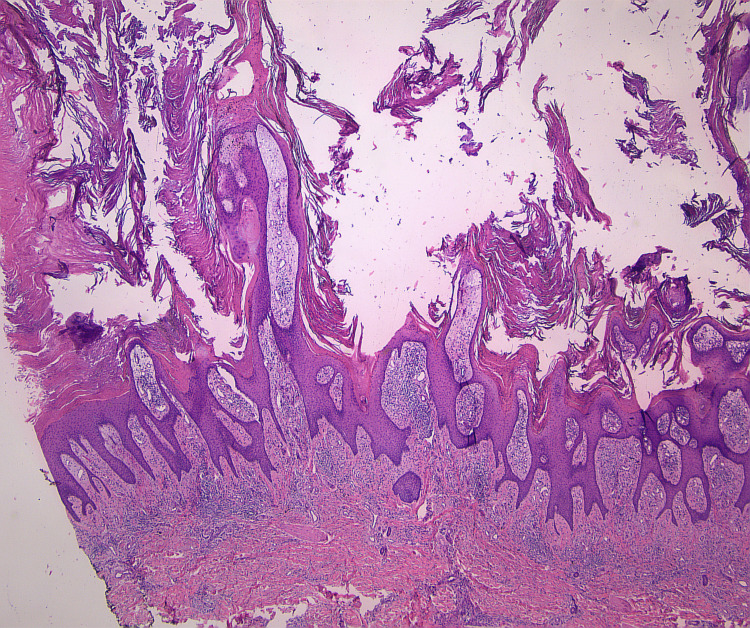
H&E stain at 2× magnification showing psoriasiform epidermal hyperplasia and extensive hyperkeratosis, with lymphocytic infiltrate near the dermal-epidermal junction

**Figure 5:**
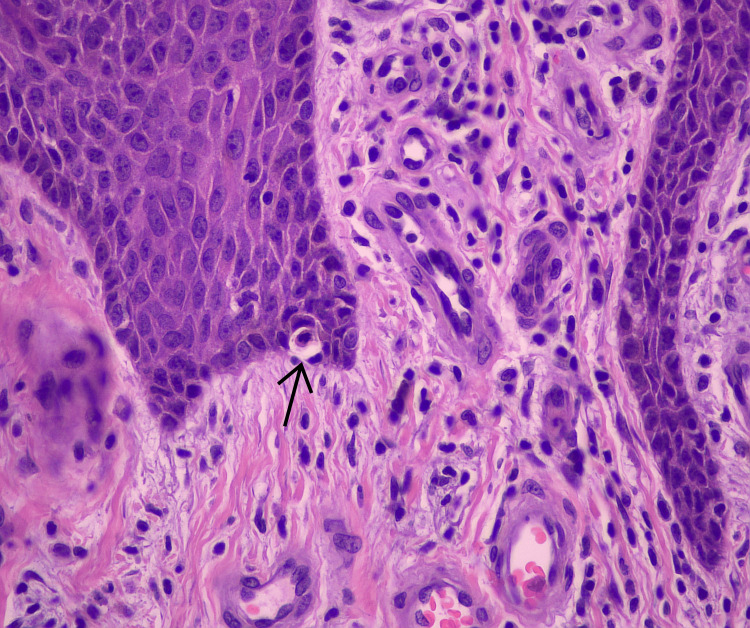
H&E stain at 40× magnification The arrow indicates an apoptotic basal keratinocyte, a characteristic histologic finding of LP. LP, lichen planus

At a follow-up visit, shave biopsies were obtained from two of the plaques (Figure 3). Histopathology revealed psoriasiform epidermal hyperplasia, a band of lymphocytic infiltration with rare dyskeratotic basal keratinocytes, and marked hyperkeratosis with zones of parakeratosis (Figure 4, Figure 5). Overall, the lesion demonstrated hypogranulosis.

Based on these findings, a diagnosis of HLP was made. The remaining plaques were partially removed at a subsequent visit, and the bases of the plaques were injected with triamcinolone (20 mg/mL) to alleviate the patient’s pruritus. The patient was prescribed 0.1% triamcinolone ointment for pruritus and 12% lactic acid ointment for her ichthyosis vulgaris. The treatment response was favorable, with relief of pruritus and a reduction in lesion size.

## Discussion

The diagnosis of HLP in this case was made by correlating clinical and histopathologic findings. Epidemiologically, HLP typically appears in middle-aged patients and is often associated with intense pruritus, as reported by the patient in this case [2,3]. Subsequent examinations revealed fine gray lines over the plaques, consistent with Wickham striae, a hallmark feature of LP. The lesions’ location on the anterior lower legs and their symmetric, bilateral distribution also corresponded with the typical pattern for HLP [1,2]. Histologically, the biopsy demonstrated focal lymphocytic infiltrates along the dermal-epidermal junction, with apoptotic basal keratinocytes, or Civatte bodies, both of which are characteristic of LP [1,2].

Although the lesions’ location and distribution were typical of HLP, their markedly exophytic morphology was unusual. We hypothesize that this abnormal presentation could be related to the patient’s underlying ichthyosis vulgaris, another hyperkeratotic condition. There is no previously reported interaction between HLP and ichthyosis vulgaris. Ichthyosis vulgaris is caused by mutations in the filaggrin gene, resulting in defective epidermal barrier function and increased keratin production as a compensatory mechanism for barrier disruption [5]. In contrast, HLP is thought to result from T-cell-mediated damage to basal keratinocytes, which induces reactive proliferation and hyperkeratosis [1]. We propose that the dryness caused by ichthyosis vulgaris may have exacerbated pruritus around the plaques, leading to increased excoriation by the patient, which in turn may have contributed to the exophytic growth pattern. Mechanical disruption is known to stimulate keratin deposition in hyperkeratotic conditions like HLP [1]. Further studies are needed to explore the potential interplay between these conditions.

Differential diagnoses included keratotic neoplasms such as keratoacanthoma, hypertrophic actinic keratosis, and SCC, as well as prurigo nodularis and severe lichen simplex chronicus. SCC was a particular concern, given the overlap in appearance with HLP [1,6]. A misdiagnosis of HLP as SCC could lead to unnecessary surgery, while the reverse would delay vital cancer treatment. Differentiating HLP from SCC can be challenging; however, irregular psoriasiform hyperplasia, wedge-shaped hypergranulosis, and hyperorthokeratosis may suggest HLP in cases of uncertainty [6]. Clinicopathologic correlation is essential, as histopathologic features may vary between cases [1,6]. Notably, SCC can develop in longstanding HLP lesions, though this was not the case in our patient. The clinical and histopathologic features in this case were more consistent with HLP, and SCC was ruled out.

Treatment for HLP typically involves topical high-potency steroids, with intralesional steroid injections used for multiple or refractory lesions [2]. The prognosis for HLP is generally good, as it is not malignant [1,2]. Surgery is not typically recommended, as it may result in poorly healing wounds, particularly in middle-aged and elderly patients [1,6]. In this case, partial excision of the lesions was performed due to their significant impact on the patient’s quality of life, as the lesions were both symptomatic and an inconvenience.

## Conclusions

The diagnosis of HLP in this case was established through the clinical appearance and location of the lesions, particularly on the anterior lower legs, and was corroborated by characteristic biopsy findings. This case underscores the importance of recognizing HLP’s clinical features and the necessity of clinicopathologic correlation for accurate diagnosis. Given the significant overlap between HLP and SCC in terms of appearance, precise differentiation is crucial to ensure timely and appropriate treatment for either condition. Of note, the unusual growth pattern of the plaques may be related to the patient’s concurrent ichthyosis vulgaris. While there is no known interaction between these two conditions, further research may uncover a potential synergistic effect on keratin overproduction, contributing to enhanced lesion growth when both disorders coexist in an individual.
